# A Comparative Study of the Microbiological Efficacy of Polymyxin B on Different Carbapenem-Resistant Gram-Negative Bacteria Infections

**DOI:** 10.3389/fmed.2021.620885

**Published:** 2021-02-09

**Authors:** Qiong Lu, Hai-Hong Zhu, Guo-Hua Li, Ting-Ting Qi, Liang-Jun Ye, Xin-Qi Teng, Qiang Qu, Ge-Fei He, Jian Qu

**Affiliations:** ^1^Department of Pharmacy, The Second Xiangya Hospital, Central South University, Institute of Clinical Pharmacy, Central South University, Changsha, China; ^2^Department of Pharmacy, Hunan Provincial Corps Hospital of Chinese People's Armed Police Force, Changsha, China; ^3^Department of Pharmacy, Xiangya Hospital, Central South University, Changsha, China; ^4^Department of Pharmacy, The First Hospital of Changsha, Changsha, China

**Keywords:** polymyxin B, carbapenem-resistant organisms, tigecycline, microbiological efficacy, bacteria clearance

## Abstract

**Objective:** The emergence of carbapenem-resistant gram-negative bacteria (CR-GNB) has brought great challenges to clinical anti-infection treatment around the world. Polymyxins are often considered as the last line of defense in the treatment of CR-GNB infections. In this study, we explored the microbiological efficacy of Polymyxin B (PMB) on different CR-GNB infections as well as the factors influencing microbiological efficacy.

**Methods:** CR-GNB infected patients with PMB-based regimens were enrolled. Clinical and microbiological data were collected from the medical electronic record system of the Second Xiangya hospital. The efficacy of PMB on different CR-GNB was evaluated by the clearance rate at 7-days and within the course of treatment, as well as the 30-day mortality rate.

**Results:** A total of 294 CR-GNB infected patients were enrolled: 154 *CR-Acinetobacter baumannii* (CRAB), 55 *CR-Klebsiella pneumoniae* (CRKP), and 85 *CR-Pseudomonas aeruginosa* (CRPA). The CRAB group had the highest 7-day bacterial clearance rate [(CRAB: 39.0%) vs. (CRKP: 29.4%) vs. (CRPA: 14.5%), *P* = 0.003] and total bacterial clearance rate [(CRAB: 49.0%) vs. (CRKP: 39.8%) vs. (CRPA: 18.2%), *P* < 0.001] among the three groups, while the bacterial clearance rate of the CRPA group was the lowest. Multivariate logistic regression showed that the differences among the three groups were multiple CR-GNB infections (*P* = 0.004), respiratory infections (*P* = 0.001), PMB resistance (*P* < 0.001), and the combination of tigecycline (*P* < 0.001). Binary logistic regression showed that multiple CR-GNB infection [(7-day bacterial clearance: P = 0.004) & (total bacterial clearance: *P* = 0.011)] and bacterial species [(7-day bacterial clearance: *P* < 0.001) & (total bacterial clearance: *P* < 0.001)] were independent risk factors for microbiological efficacy.

**Conclusion:** PMB exhibited differential microbiological efficacy on different types of CR-GNB infections; it had the best effect on CRAB, followed by CRKP and CRPA. Multiple CR-GNB infections and bacterial species were independent risk factors for microbiological efficacy.

## Introduction

Carbapenem-resistant gram-negative bacteria (CR-GNB) infections are an urgent global public health threat with high mortality rate (1). According to the standard of Clinical and Laboratory Standards Institute (CLSI), minimum inhibitory concentrations (MICs) of both imipenem and meropenem ≥8 mg/L means carbapenem-resistance, and MIC ≥4 mg/L means intermediate susceptibility (2). However, in the new guide of the European Committee on Antimicrobial Susceptibility Testing (EUCAST), the breakpoints of imipenem and meropenem are 4 and 8mg/L, respectively (3). Carbapenem-resistant Enterobacteriaceae (CRE), Pseudomonas, and Acinetobacter species are the three most common CR-GNB (4, 5). The emergence of these bacteria has brought great challenges to clinical anti-infection treatment around the world, and rational selection of antibacterial drugs has become an important way to prevent and treat CR-GNB infections (6). There are many clinical studies and summaries regarding anti-CR-GNB drugs such as polymyxins (7), tigecycline (8), and ceftazidime-avibactam (9).

Polymyxins, a kind of polypeptide antibiotic, are often regarded as the last line of defense in the treatment of CR-GNB infections, and polymyxin B (PMB) and colistin are the two drugs under heated discussion. A system review and meta-analysis showed that being elderly, high daily dose, having underlying diseases such as diabetes, and use of concomitant nephrotoxic drugs were independent predictors of polymyxin-induced nephrotoxicity (10). A recent cohort study also showed that high-dose PMB was associated with increased nephrotoxicity (11). Therefore, therapeutic drug monitoring should be done to outweigh the potential benefits of polymyxin therapy from its risk (10).

Some studies have explored the efficacy of PMB on a specific bacterial infection of CR-GNB (either CRAB or CRKP), but no studies have compared the efficacy of PMB for three different types of CR-GNB infection. PMB has been shown to have a strong bactericidal effect on carbapenem-resistant *Acinetobacter baumannii* (CRAB) *in vitro* (12). Sun et al. indicated that even with salvage use of PMB, the tigecycline based medications did not significantly increase the 28-day mortality rate of patients infected with carbapenem-resistant *Klebsiella pneumoniae* (CRKP) (13). There is also evidence to suggest that early use of PMB can reduce the mortality of CRKP bloodstream infections (BSIs) (14). Moreover, the combination of PMB and imipenem presented synergistic antibacterial effects against carbapenem-resistant *Pseudomonas aeruginosa* (CRPA) *in vitro* (15). Although PMB has curative effects on many types of CR-GNB infections, its differential efficacy with respect to various bacteria has not been clearly discussed. In this study, we explored the efficacy of PMB on different CR-GNBs and factor influencing efficacy.

## Patients and Methods

### Ethical Approval

This research was approved by the Ethics Committees of the Second Xiangya Hospital of Central South University in Changsha, China (LYF-2020021). The implementation of this study was in line with the Declaration of Helsinki and its amendments. Informed consent was waived because this study was non-interventional in nature.

### Patients

Data were collected concerning patients with PMB medication histories in the Second Xiangya Hospital of Central South University from January 1, 2018 to March 31, 2020. The inclusion criteria were as follows: (1) Patients treated with PMB (Shanghai Number 1 Biochemical & Pharmaceuticals, Shanghai, China) for CR-GNB infection; (2) The duration of PMB treatment was not <72 h; (3) CR-GNB infection was confirmed by culture results and drug sensitivity test results were available. The exclusion criteria were as follows: (1) The number of patients infected with certain types of CR-GNB was too small to perform inferential analyses; (2) Patients with malignant tumors or severe liver/kidney dysfunction before treatment; (3) Incomplete clinical data.

### Collection of Clinical Data

We collected the relevant information of patients that may be related to the efficacy of PMB, including basic demographic information, medication (treatment duration, dosage, frequency, and combined drugs), diagnoses, pathogenic bacteria (species, sensitivity, and site of infection), Acute Physiology and Chronic Health Evaluation (APACHE) II scores, efficacy evaluation indices and prognoses. “Single CR-GNB” represents infection caused by one CR-GNB; “multiple CR-GNB” represents infection caused by two or more CR-GNB.

### Research Design

According to the types of CR-GNB infection, the patients were divided into three groups: CRAB, CRKP, and CRPA infections. Patients infected with multiple CR-GNB before and during the treatment course were split into different cases according to the type of bacteria, and then assigned to the appropriate group. The microbiological efficacy of PMB on different bacteria was evaluated by the bacterial clearance rate after the course of treatment (total bacterial clearance rate) and the 7-day bacterial clearance rate. Mortality was defined as deaths in hospitals or the discontinuation of treatment in severe cases due to poor outcomes.

### Microbiology

The sensitivity of bacteria to antibacterial drugs was tested by the broth-microdilution method using analytical instruments. MIC was determined by a VITEK®2 system (bioMérieux, Marcy-l'Étoile, France) based on the recommendations of the EUCAST. “Carbapenem resistance” was defined as the MIC of bacteria to imipenem and meropenem ≥4 mg/L. Meanwhile, according to the European Committee on Antimicrobial Susceptibility Testing (EUCAST, v8.0, 2018), “MIC >2 mg/L” represented bacterial resistance to tigecycline and PMB (16).

### Statistical Analysis

Before the start of the study, the appropriate sample size was estimated by the following equation: N = Z^2^P (1 – P)/E^2^ (*Z* = 1.96; *P* = 0.5; *E* = 10%), and the result showed that the minimum sample size is 96. Data were analyzed using SPSS v21.0 (IBM, Armonk, NY, USA). Data conforming to the normal distribution were analyzed by *t*-tests or analysis of variance (ANOVA) and expressed as mean ± standard deviation. Non-normally distributed data were analyzed by nonparametric tests and expressed by median and interquartile range (IQR). Count data were analyzed by the chi-square tests and expressed by quantity and percentage. In multiple groups analysis, measurement data were analyzed by one-way analysis of variance (ANOVA) or non-parametric test, and the *post-hoc* test (LSD) was further used for pairwise comparisons; count data were analyzed by chi-square test for pairwise comparisons. The binary logistic regression used the entry method, and *P* < 0.05 was considered statistically significant. In addition, multiple logistic regression was used to analyze data with multiple endpoints. Cox-regression analysis was applied to compare the 30-day mortality of the three types of CR-GNB infected patients and influencing factors, and *P* < 0.05 was considered statistically significant.

## Results

### Characteristics

According to the inclusion and exclusion criteria, 294 CR-GNB infected patients were enrolled: 154 (52.4%) CRAB strains, 55 (18.4%) CRKP strains, and 85 CRPA strains. Nine patients were infected with carbapenem-resistant *Escherichia coli* and *Enterobacter cloacae*, and were excluded due to insufficient numbers. The clinical characteristics are shown in Table 1. The average age of enrolled patients was 57.32 ± 19.29 years and the majority (72.4%) were male. Patients with mechanical ventilation and vasoactive agents accounted for 73.1 and 52.7% of the sample, respectively. The ICU admission rate was 71.4%. The most common infection site was the respiratory tract (88.4%), and 86.0% of patients suffered from the respiratory system diseases. The average dose of PMB was 0.86 (0.83–1.00) mg/kg/q12h and the average course of treatment was 11.75 (7.50–15.88) days. 5.1% of pathogenic bacteria were PMB resistant, and the total bacterial clearance rate was 40.4%, while the 7-day bacterial clearance rate was 31.6%. Tigecycline was the most frequently used drug in the combination with PMB (33.4%). 28.2% of patients died in hospital or discontinued treatment due to poor efficacy.

**Table 1:** Clinical characteristics of CR-GNB infected patients with PMB-based regimens.

**Parameters**	**Patients (*n* = 294)**
Age	57.32 ± 19.29
Male	213 (72.4%)
Weight (kg)	60.00 (50.00–60.00)
Mechanical ventilation	215 (73.1%)
Vasoactive agents	155 (52.7%)
APACHE II score	20.49 ± 8.50
**Source of infection**	
Respiratory tract	260 (88.4%)
Blood	70 (23.8%)
Urinary tract	28 (9.5%)
Central nervous system	14 (4.7%)
Abdomen	23 (7.8%)
**Underlying disease**	
Respiratory system	253 (86.0%)
Cardiovascular	190 (64.6%)
Diabetes	65 (22.1%)
Liver damage	78 (26.5%)
Kidney damage	136 (46.2%)
Digestive system	57 (19.4%)
**Drug sensitivity**	
**Tigecycline**	
MIC >2 (mg/L)	267 (92.7%)
**PMB (PMB)**	
MIC ≤0.5 (mg/L)	107 (36.4%%)
MIC ≤1 (mg/L)	169 (57.5%)
MIC >2 (mg/L)	15 (5.1%)
**Combination**	
Tigecycline	98 (33.4%)
Carbapenems	78 (26.5%)
β-lactams	97 (33.0%)
Glycopeptides	45 (15.3%)
Treatment duration (days)	11.75 (7.50–15.88)
Average dose (mg/kg/q12h)	0.86 (0.83–1.00)
ICU admission (n, %)	210 (71.4%)
Hospitalization length (days)	39.00 (24.00–69.25)
7-day bacterial clearance rate (n, %)	93 (31.6%)
Bacterial clearance rate (n, %)	116 (40.4%)
Bacterial clearance time (days)	7.00 (5.00–12.00)
Mortality rate (n, %)	83 (28.2%)
Survival time (days)	9.00 (6.00–13.50)

*Bacterial clearance rate, the rate that the bacteria were eliminated during the course of treatment; Mortality rate, the rate that patients died in the hospital or discontinued treatment due to poor efficacy*.

### Microbiological Efficacy in Different CR-GNB Infection Contexts

We compared the characteristics and clinical parameters among the three groups. The results showed that patients in the CRPA group had a higher average age than the other two groups [(CRAB: 56.83 ± 19.60 years) vs. (CRKP: 54.09 ± 19.93 years) vs. (CRPA: 63.67 ± 15.94 years), *P* = 0.014]. In the CRKP group, the proportions of patients who were male [(CRAB: 77.3%) vs. (CRKP: 62.4%) vs. (CRPA: 74.5%), *P* = 0.044] and exhibited respiratory tract infection [(CRAB: 96.8%) vs. (CRKP: 70.6%) vs. (CRPA: 92.7%), *P* < 0.001] were lower than the other two groups.

There were statistically significant differences in the resistance of the three bacteria to antimicrobial drugs: CRPA was naturally resistant to tigecycline, while CRAB was more resistant to tigecycline than CRKP [(CRAB: 94.7%) vs. (CRKP: 84.1%), *P* = 0.007]. In addition, CRPA was more resistant to PMB (*P* < 0.001). Furthermore, compared with the other two groups, tigecycline was used less frequently to treat CRPA infection [(CRAB: 33.4%) vs. (CRKP: 44.0%) vs. (CRPA: 14.5%), *P* = 0.001]. More CRPA infections were accompanied by other CR-GNB [(CRAB: 33.1%) vs. (CRKP: 44.7%) vs. (CRPA: 54.5%), *P* = 0.013].

**Figure 1:**
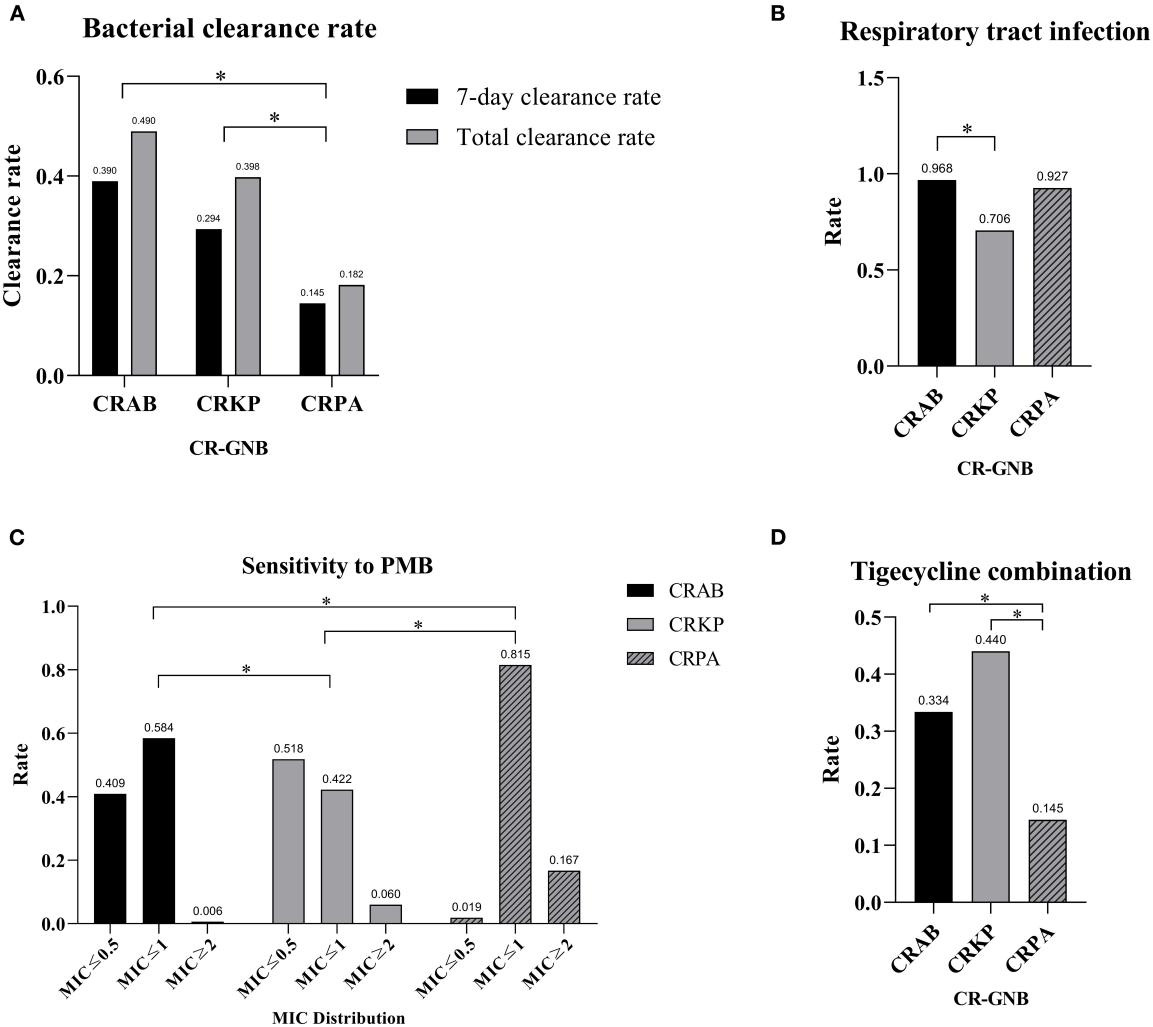
Divergences among different CR-GNB groups. **(A)** Divergence in clearance rates. **(B)** Divergence in respiratory tract infection rates. **(C)** Divergence in PMB resistance rates. **(D)** Divergence in the tigecycline combination rate. *p < 0.05.

The CRAB group had the highest 7-day bacterial clearance rate [(CRAB: 39.0%) vs. (CRKP: 29.4%) vs. (CRPA: 14.5%), *P* = 0.003] and total bacterial clearance rate [(CRAB: 49.0%) vs. (CRKP: 39.8%) vs. (CRPA: 18.2%), *P* < 0.001] among the three groups, while the bacterial clearance rate of the CRPA group was the lowest. There was no statistical difference among the three groups in terms of treatment duration and mortality rate (Table 2 and Figure 1A). Next, we performed multiple logistic regression on the three CR-GNB in order to control for the interference of confounding factors, and the results showed that the differences among them were multiple CR-GNB infection (*P* = 0.004), respiratory infection (*P* = 0.001), PMB resistance (*P* < 0.001), and the combination of tigecycline (*P* < 0.001) (Table 3 and Figures 1B–D).

**Table 2:** Characteristics comparison of patients infected with different CR-GNB.

**Parameters**	**CRAB (*n* = 154)**	**CRKP (*n* = 85)**	**CRPA (*n* = 55)**	***P***	Pairwise comparison*		
					**P_**12**_**	**P_**13**_**	**P_**23**_**
Age	56.83 ± 19.60	54.09 ± 19.93	63.67 ± 15.94	**0.014**	0.288	**0.023**	**0.004**
Male	119 (77.3%)	53 (62.4%)	41 (74.5%)	**0.044**	**0.014**	0.682	0.134
Weight (kg)	60.00 (50.00–60.00)	57.50 (50.00–60.00)	60.00 (49.00–60.00)	0.454	0.265	0.370	0.936
Mechanical ventilation	117 (76.0%)	59 (69.4%)	39 (70.9%)	0.504	0.270	0.459	0.850
Vasoactive agents	84 (54.5%)	41 (48.2%)	30 (54.5%)	0.617	0.350	1.000	0.466
APACHE II score	20.76 ± 8.24	20.25 ± 9.35	20.08 ± 8.17	0.891	0.724	0.677	0.927
**Source of infection**							
Respiratory tract	149 (96.8%)	60 (70.6%)	51 (92.7%)	**<0.001**	**0.050**	0.293	0.561
Blood	35 (22.7%)	24 (28.2%)	11 (20.0%)	0.483	0.344	0.675	0.272
Urinary tract	10 (6.5%)	12 (14.1%)	6 (10.9%)	0.146	0.051	0.290	0.580
Central nervous system	11 (7.1%)	3 (3.5%)	0 (0.0%)	0.084	0.255	**0.042**	0.159
Abdomen	9 (5.8%)	10 (11.8%)	4 (7.3%)	0.260	0.105	0.707	0.387
**Underlying disease**							
Respiratory system	135 (87.7%)	69 (81.2%)	49 (89.1%)	0.295	0.175	0.779	0.209
Cardiovascular	97 (63.0%)	57 (67.1%)	36 (65.5%)	0.812	0.529	0.744	0.844
Diabetes	29 (18.8%)	21 (24.7%)	15 (27.3%)	0.342	0.285	0.187	0.734
Liver	43 (27.9%)	20 (23.5%)	15 (27.3%)	0.655	0.461	0.926	0.617
Kidney	63 (40.9%)	46 (54.1%)	27 (49.1%)	0.755	**0.050**	0.293	0.107
Digestive system	29 (18.8%)	15 (17.6%)	13 (23.6%)	0.342	0.803	0.458	0.387
Sensitivity							
Tigecycline	143 (94.7%)	69 (84.1%)	ND		**0.007**		
MIC >2 (mg/L)							
PMB							
MIC ≤0.5 (mg/L)	63 (40.9%)	43 (51.8%)	1 (1.9%)	**<0.001**	**0.006**	**<0.001**	**<0.001**
MIC ≤1 (mg/L)	90 (58.4%)	35 (42.2%)	44 (81.5%)				
MIC ≥2 (mg/L)	1 (0.6%)	5 (6.0%)	9 (16.7%)				
Multiple CR-GNB infection	51 (33.1%)	38 (44.7%)	30 (54.5%)	**0.013**	0.076	**0.005**	0.255
**Combination**							
Tigecycline	53 (33.4%)	37 (44.0%)	8 (14.5%)	**0.001**	0.143	**0.005**	**<0.001**
Carbapenems	38 (24.7%)	28 (32.9%)	12 (21.8%)	0.260	0.171	0.670	0.155
β-lactams	52 (33.8%)	24 (28.2%)	21 (38.2%)	0.453	0.379	0.555	0.218
Glycopeptides	26 (16.9%)	11 (12.9%)	8 (14.5%)	0.709	0.420	0.687	0.787
Treatment duration (days)	11.00 (7.00–15.00)	12.25 (8.00–17.00)	10.00 (7.00–16.00)	0.514	0.286	0.889	0.364
Average dose (mg/kg/q12h)	0.86 (0.83–1.00)	0.87 (0.82–1.00)	0.84 (0.81–1.00)	0.615	0.614	0.343	0.607
ICU (n, %)	117 (76.0%)	57 (67.1%)	36 (65.5%)	0.191	0.138	0.131	0.844
Hospitalization length (days)	37.00 (21.00–65.50)	46.00 (24.00–78.50)	38.00 (28.00–67.00)	0.122	**0.039**	0.472	0.391
7-day bacterial clearance rate (n, %)	60 (39.0%)	25 (29.4%)	8 (14.5%)	**0.003**	0.140	**0.001**	**0.043**
Total bacterial clearance rate (n, %)	73 (49.0%)	33 (39.8%)	10 (18.2%)	**<0.001**	0.176	**<0.001**	**0.007**
Clearance time (days)	7.00 (4.00–7.00)	10.00 (5.50–14.00)	8.00 (5.00–13.00)	0.129	**0.048**	0.458	0.422
Mortality rate (n, %)	47 (30.5%)	20 (23.5%)	16 (29.1%)	0.501	0.249	0.843	0.462
Lifetime (days)	8.00 (5.00–12.00)	8.50 (5.50–18.25)	11.50 (7.50–47.00)	0.371	0.746	0.172	0.301

*P_12_, P_13_, and P_23_ denote the pairwise comparison of CRAB and CRKP, CRAB and CRPA, CRKP and CRPA, respectively. ND, not done (because CRPA is naturally resistant to tigecycline, antimicrobial susceptibility testing was not carried out). Statistically significant differences are emboldened*.

**Table 3:** Multivariate logistic regression with CR-GNB type as the classification indicator.

**Factors**	**χ^2^**	***P***
Sex	5.379	0.068
Age	131.443	0.399
Multiple CR-GNB infection	11.276	**0.004**
Respiratory tract infection	13.468	**0.001**
Sensitivity to PMB	44.963	**<0.001**
Tigecycline combination	17.176	**<0.001**

*Results with statistical differences are indicated in bold*.

### Factors Influencing Microbiological Efficacy

The above results indicated that age, gender, multiple CR-GNB infection, respiratory tract infection, sensitivity to PMB, and the combination of tigecycline may be associated with the different clearance rates for different CR-GNB. However, whether these factors can cause differences in bacterial clearance rates still needs to be verified. Therefore, we performed a binary logistic regression and found that multiple CR-GNB infection [(7-day bacterial clearance: *P* = 0.004) & (total bacterial clearance: *P* = 0.011)] and bacterial species [(7-day bacterial clearance: *P* < 0.001) & (total bacterial clearance: *P* < 0.001)] were independent risk factors for bacterial clearance (Table 4).

**Table 4:** Binary logistic regression with 7-day bacterial clearance.

**Endpoint**	**Factors**	**B**	***P***	**Exp (B)**	**Lower**	**Upper**
7-day bacterial clearance	Age	0.006	0.380	1.006	0.992	1.021
	Sex	−0.382	0.204	0.682	0.378	1.231
	Respiratory tract infection	−0.137	0.770	0.872	0.349	2.177
	Multiple CR–GNB infection	0.795	**0.004**	**2.214**	**1.289**	**3.801**
	Sensitivity to PMB	0.327	0.197	1.387	0.844	2.280
	Tigecycline	−0.496	0.100	0.609	0.337	1.100
	Bacteria species	−0.840	**<0.001**	**0.432**	**0.291**	**0.640**
Total bacterial clearance	Age	0.002	0.821	1.002	0.988	1.015
	Sex	−0.166	0.566	0.847	0.480	1.494
	Respiratory tract infection	−0.223	0.603	0.800	0.345	1.857
	Multiple CR–GNB infection	0.675	**0.011**	**1.964**	**1.167**	**3.306**
	Sensitivity to PMB	−0.016	0.946	0.984	0.612	1.581
	Tigecycline	−0.403	0.153	0.669	0.385	1.162
	Bacteria species	−0.788	**<0.001**	**0.455**	**0.314**	**0.658**

*Assignment: 1 = Acinetobacter baumanni, 2 = Klebsiella pneumoniae, 3 = Pseudomonas aeruginosa; 0 = sensitive, 1 = resistant. Statistically significant differences are emboldened*.

In order to clarify the effect of multiple CR-GNB infection on bacterial clearance, we divided the cases into two groups “Single CR-GNB” and “Multiple CR-GNB,” and compared the treatment information and outcomes of the two groups. The results showed that the medication information between the two groups was well-matched, and cases of multiple CR-GNB infection did not cause a statistical difference in clearance rate (Supplementary Table 1). Furthermore, we compared the clinical data of patients with and without bacterial clearance at 7 days and at the end of the PMB treatment course. The results showed that the above factors did not significantly affect the respective clearance rates (Supplementary Table 2).

### Cox-Regression Survival Analysis for 30-Day Mortality

**Figure 2:**
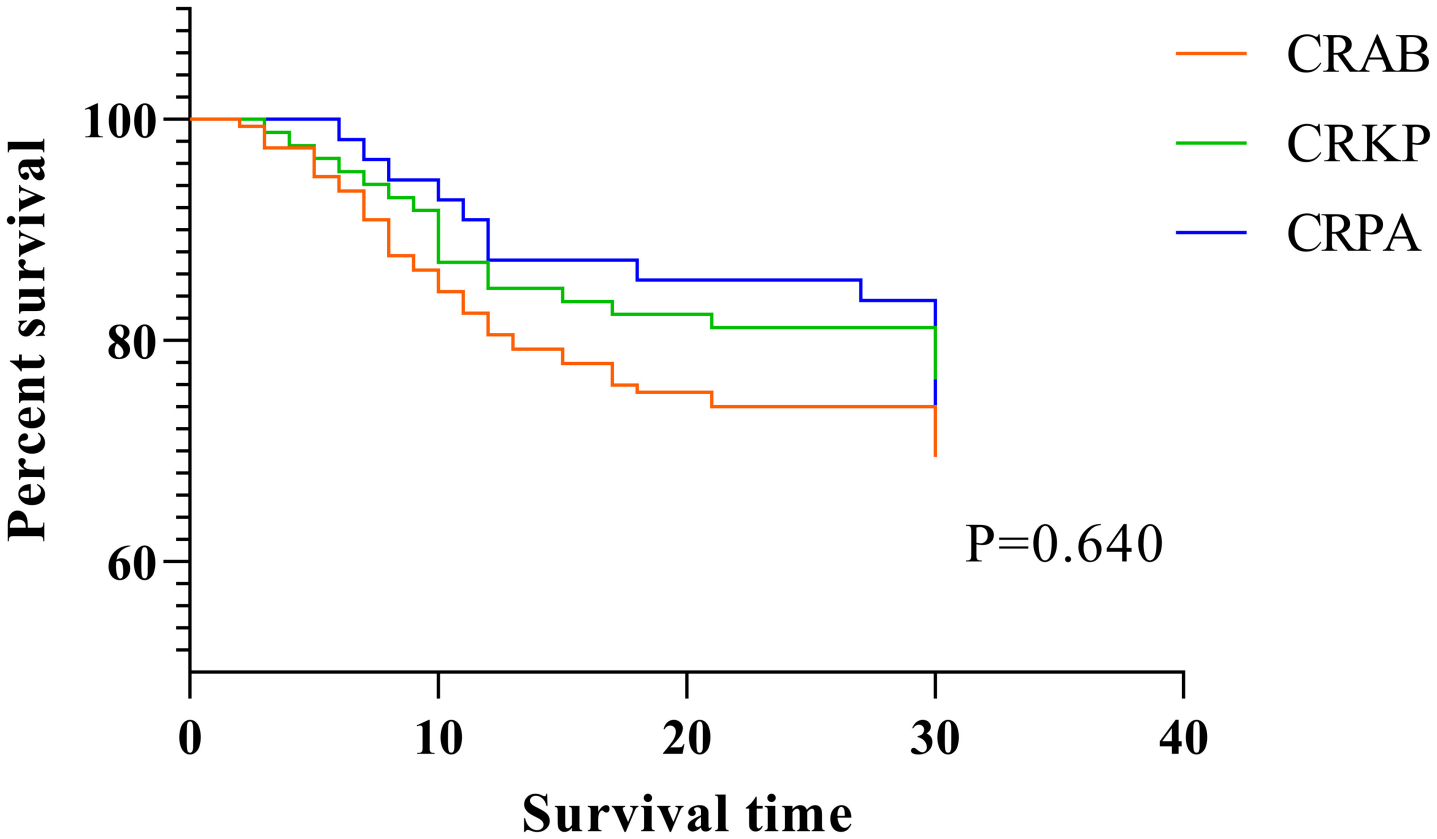
Cox-regression analysis for 30-day mortality.

Finally, in order to compare the 30-day mortality rate and survival time among the three CR-GNB groups, we performed Cox-regression survival analysis and found that none of the above factors significantly affected the 30-day mortality or survival time statistically (Table 5 and Figure 2).

**Table 5:** Cox-regression analysis for 30-day mortality.

**Variable**	***P***	**Hazard ratio**	**95% CI**	
			**Lower limit**	**Upper limit**
Sensitivity to PMB	0.272	0.788	0.515	1.206
Multiple CR-GNB infection	0.365	0.810	0.513	1.278
Tigecycline combination	0.288	1.288	0.808	2.055
Respiratory tract infection	0.582	1.265	0.547	2.927
Bacteria species	0.640			
CRAB	0.371	0.755	0.443	1.355
CRKP	0.910	1.038	0.544	1.980

*The survival analysis for different bacteria species used CRPA as a control*.

## Discussion

CR-GNB infections pose great challenges to clinical treatment (17). PMB is an important drug for the treatment of CR-GNB infections, but its microbiological efficacy against different pathogens still needs to be elucidated. In this study, we explored the microbiological efficacy of PMB on CRAB, CRKP, and CRPA. We also explored the factors influencing PMB microbiological efficacy and 30-days mortality. The results showed that PMB has different microbiological efficacy among CR-GNBs, with the best in CRAB, followed by CRKP and CRPA. Moreover, multivariate logistic regression showed that the differences among the three groups were multiple CR-GNB infection, respiratory infection, PMB resistance, and the combination of tigecycline. Multiple CR-GNB infection and bacterial species may be risk factors for the microbiological efficacy of PMB-based regimens.

We found that it was easier to eliminate CRAB by PMB compared to CRKP and CRPA. Previous studies have also shown that PMB has good efficacy on respiratory tract infections caused by *Acinetobacter baumannii, Pseudomonas aeruginosa*, and multidrug-resistant Gram-negative bacteria (18). Another study which carried out a Cox regression with 30-day mortality as the endpoint showed that the PMB-based regimen is beneficial to lower respiratory tract infections and *Acinetobacter baumannii* infections compared with *Pseudomonas aeruginosa* infections, which is consistent with our results (19). In terms of PMB combinations, a checkerboard assay study found that PMB plus sulbactam exhibited the highest synergistic effect at a rate of 82.35% in CRAB infections (20). Further research is needed to explore which combinations are the most effective against CRAB infection.

In our study, CRPA was naturally resistant to tigecycline and exhibited the highest resistance to PMB, which may be attributed to the production of broad-spectrum β-lactamase and metallo-β-lactamase (21). CRPA is inherently resistant to a variety of antibacterial drugs; it can develop resistance easily through multiple mechanisms (22). The PMB resistance rate is <5% globally, but it is increasing gradually and even approaches 50% in Singapore (23). These characteristics make it difficult for PMB to clear CRPA successfully (17). Because CRPA is naturally resistant to tigecycline, combining tigecycline, and PMB in this context exhibited the lowest efficacy; therefore, the purpose of such a combination may be to treat coexisting CR-GNB infections. Since PMB monotherapy commonly leads to the occurrence of drug resistance, it is necessary to select the appropriate combination drugs. Studies have shown that PMB combined with tigecycline shows promising rapid and long-lasting bactericidal effects in CRAB and CRE including CRKP, both *in vivo* and *in vitro* (24–26). Treatment with 100 mg PMB q12h plus 200 mg tigecycline q12h significantly reduced bacterial density (27). Finding ways to improve the clearance rate and efficacy of CRPA infection is an urgent clinical problem. An *in vitro* study indicated that the combination of PMB and enrofloxacin can work synergistically against CRPA (28). Moreover, PMB and usnic acid have also been shown to exhibit a synergistic effect against CRPA infection (29). These existing findings may provide an important benchmark for finding more effective PMB combinations.

A single-center retrospective study showed that receiving PMB-based therapy provided a survival benefit compared with tigecycline-based therapy in the context of CRKP bloodstream infection (30). Time-lapse microscopy and time-kill experiments evaluated PMB in combination with 13 other antibiotics against CRKP and found that PMB in combination with minocycline, rifampicin, or fosfomycin could be of potential clinical interest (31). The 7-days bacterial clearance rate and total bacterial clearance of CRKP in our study were 29.4 and 39.8%, respectively; the microbiological efficacy of PMB in CRKP is lower than that in CRAB and higher than that in CRPA.

*In vitro* and *in vivo* experiments of different types of CR-GNB with different combinations based on PMB are worthy of further study. Different combination regimens may have different clinical and microbial effects on different bacteria. Therefore, joint drug sensitivity experiments are a useful tool for investigating the combined bactericidal effects of various PMB combined regimens *in vitro*. Because of our limited sample size, we did not carry out combination regimens subgroups analyses. Other research has investigated this important issue (32, 33). One study evaluated the activity of ceftazidime-avibactam and PMB in combination against CRKP in a tandem *in vitro* time-kill/*in vivo* Galleria mellonella survival model assay and found no improvement in *in vitro* bactericidal activity or *in vivo* efficacy using this combination (33). Another investigation focused on 82 KPC-KP BSIs and found that PMB plus amikacin showed a survival benefit compared with other regimens (32). Moreover, with the development of new technologies, methods such as machine learning brings forth a possible avenue to optimize treatment regimens beyond the use of the “traditional” indices of antibiotic action (34). This methodology leverages *in vitro* experimental data, a mathematical pharmacodynamic model, and population pharmacokinetics to optimize antibiotic combinations (34). For precise treatment, therapeutic drug monitoring (TDM) guided medication of PMB has also been used in the clinical practice to ensure efficiency and safety (35).

Beyond the individual types of CR-GNB, multiple CR-GNB infection and bacterial species may be risk factors for microbiological efficacy of PMB-based regimens. While in the separate analysis of CR-GNB and the binary logistic regression analysis, no exact factors were found that may lead to different clearance rates, and the divergences in clearance rates failed to lead to differences in survival time according to the results from Cox regression. These results also suggested that there may be internal factors associated with different CR-GNB that make clearance difficult.

There were some limitations to our study. First, this single center retrospective study with a limited sample size needs more cases to drawn robust conclusions. Second, the clinical data on patients were not detailed enough, and the dosage information of combined drugs was not comprehensive. In addition, the number of CR-GNB subgroups is small.

In conclusion, our study firstly explored the microbiological efficacy of PMB on different CR-GNB infections and the factors influencing microbiological efficacy. The results showed that PMB was an effective antibiotic in the treatment of CR-GNB infection, with differential efficacy depending on the type of CR-GNB. The best effect occurred with respect to CRAB, followed by CRKP and CRPA. Multiple CR-GNB infection and bacterial species were independent risk factors for microbiological efficacy. Large-sample multicenter studies are needed to find precise strategies to optimize the microbiological efficacy of PMB on CR-GNB.

## Data Availability Statement

The original contributions presented in the study are included in the article/Supplementary Material, further inquiries can be directed to the corresponding author.

## Ethics Statement

The studies involving human participants were reviewed and approved by the Ethics Committees of the Second Xiangya Hospital of Central South University in Changsha, China (LYF-2020021). Written informed consent for participation was not required for this study in accordance with the national legislation and the institutional requirements.

## Author Contributions

JQ and QL conceptualized and designed the study. H-HZ, G-HL, T-TQ, L-JY, X-QT, QQ, and G-FH collected data. G-HL and T-TQ analyzed the data. G-HL, JQ, and QL drafted the manuscript. H-HZ modified relevant contents including language and initial draft responding to reviewers. All authors contributed to the revision and approved the final version of the manuscript.

## Conflict of Interest

The authors declare that the research was conducted in the absence of any commercial or financial relationships that could be construed as a potential conflict of interest.
