# Supplementary material for: A Comparative Study of the Microbiological Efficacy of Polymyxin B on Different Carbapenem-Resistant Gram-Negative Bacteria Infections
Source: Front Med (Lausanne). 2021 Feb 9;8:620885. doi: 10.3389/fmed.2021.620885 (PMC7902010; doi:10.3389/fmed.2021.620885)
Supplement: Supplementary file 1 [file Table_1.DOCX]

**Table S1.** Comparison of characteristics of patients infected with single CRO or multiple CR-GNB infection.

| **Parameters** | **Single CR-GNB**  **(n=175)** | **Multiple CR-GNB**  **(n=119)** | **P** |
| --- | --- | --- | --- |
|  |  |  |  |
| Age | 59.0(47.0-71.0) | 57.0(48.0-72.0) | 0.944 |
| Male | 122(69.7%) | 91(76.5%) | 0.203 |
| Weight (kg) | 56.0(50.0-60.0) | 60.0(53.0-60.0) | **0.049** |
| Mechanical ventilation | 124(70.9%) | 91(76.5%) | 0.287 |
| Vasoactive agents | 100(57.1%) | 55(46.2%) | 0.066 |
| APACHE II score | 20.0(14.0-27.0) | 21.5(18.0-25.8) | 0.488 |
| Source of infection |  |  |  |
| Respiratory tract | 156(89.1%) | 104(87.4%) | 0.646 |
| Blood | 41(23.4%) | 29(24.4%) | 0.852 |
| Urinary tract | 15(8.6%) | 13(10.9%) | 0.500 |
| Central nervous system | 7(4.0%) | 7(5.9%) | 0.457 |
| Abdomen | 13(7.4%) | 10(8.4%) | 0.760 |
| Underlying disease |  |  |  |
| Respiratory system | 152(86.9%) | 101(84.9%) | 0.630 |
| Cardiovascular | 112(64.0%) | 78(65.5%) | 0.785 |
| Diabetes | 33(18.9%) | 32(26.9%) | 0.103 |
| Liver | 42(24.0%) | 36(30.3%) | 0.233 |
| Kidney | 75(42.9%) | 61(51.3%) | 0.156 |
| Digestive system | 35(20.1%) | 22(18.5%) | 0.730 |
| Sensitivity to PMB |  |  |  |
| MIC ≤0.5 (mg/L) | 70(40.5%) | 37(31.4%) | 0.058 |
| MIC ≤1 (mg/L) | 92(53.2%) | 78(66.1%) |  |
| MIC ≥2 (mg/L) | 11(6.4%) | 3(2.5%) |  |
| Combination |  |  |  |
| Tigecycline | 56(32.2%) | 42(35.3%) | 0.579 |
| Carbapenems | 47(26.9%) | 31(26.1%) | 0.878 |
| β-lactams | 61(34.9%) | 36(30.3%) | 0.410 |
| Glycopeptides | 30(17.1%) | 15(12.6%) | 0.289 |
| Treatment duration (days) | 10.0(6.5-14.0) | 13.0(8.5-16.5) | **<0.001** |
| Average dose (mg/kg/q12h) | 0.87(0.80-1.00) | 0.83(0.83-1.00) | 0.942 |
| ICU (n,%) | 111(63.4%) | 99(83.2%) | **<0.001** |
| Hospitalization length (days) | 34.0(21.0-54.0) | 56.0(30.0-78.0) | **<0.001** |
| 7-day clearance rate (n,%) | 48(27.4%) | 45(37.8%) | 0.060 |
| Total clearance rate (n,%) | 63(36.8%) | 53(45.7%) | 0.134 |
| Clearance time (days) | 8.0(5.0-12.0) | 7.0(4.0-14.0) | 0.778 |
| Mortality rate (n,%) | 52(29.7%) | 31(26.1%) | 0.493 |
| Lifetime (days) | 30.0(30.0-30.0) | 30.0(30.0-30.0) | 0.174 |

“Single CR-GNB” represents infection caused by one CR-GNB, “Multiple CR-GNB” represents infection caused by two or more CR-GNB. Statistically significant differences are emboldened.

**Table S2.** Comparison of CR-GNB medication information with 7-day bacterial clearance or clearance in treatment duration as the endpoint.

| CR-GNB | 7-day clearance | Clearance in treatment duration |
| --- | --- | --- |
| CRAB | Urinary tract infection (**P=0.048**) | Urinary tract infection (**P=0.016**) |
| CRKP | Urinary tract infection (**P=0.035**) | Vasoactive agents (**P=0.028**)  Treatment duration (**P=0.019**) |
| CRPA | None | None |

The statistical content was the same as the basic information and medication information in **Table 2**. We only showed the results with statistical differences.
